# Biobased PEF/PLA Bilayer Electrospun Films with Enhanced Mechanical, Barrier, and UV-Protective Properties

**DOI:** 10.1021/acsomega.6c02615

**Published:** 2026-06-15

**Authors:** Rabia Zia, Bismi Phasaludeen, Anuj Niroula, Juraij Kandiyil, Ahmad Rabbani, Salahdein Aburuz, Akmal Nazir, Muhammad Z. Iqbal

**Affiliations:** † Department of Pharmacology & Therapeutics, College of Medicine and Health Sciences, 11239 United Arab Emirates University, Al Ain, PO Box 15551, United Arab Emirates; ‡ Department of Food Science, College of Agriculture and Veterinary Medicine, United Arab Emirates University, Al Ain, PO Box 15551, United Arab Emirates; § Department of Chemical & Petroleum Engineering, College of Engineering, United Arab Emirates University, Al Ain, PO Box 15551, United Arab Emirates

## Abstract

Poly­(lactic acid) (PLA) electrospun films are attractive for biomedical and packaging uses, but they are brittle and provide weak UV protection. Poly­(ethylene furanoate) (PEF) is biobased and offers excellent barrier properties but limited ductility. We fabricated PEF/PLA bilayer electrospun films in which curcumin-loaded PLA serves as the active top layer and PEF acts as a mechanically supportive, barrier, and photoprotective backing. Sequential electrospinning was adopted after identifying hexafluoro-2-propanol (HFIP) as a suitable PEF solvent that yields strong adhesion, whereas trifluoroacetic acid (TFA) produced unstable interfaces. Bilayers showed smaller, more uniform fibers, significant interfacial cohesion, and mechanical gains compared with a PLA monolayer, including higher tensile strength, elongation, and modulus. FTIR indicated no new covalent bonds on curcumin addition, consistent with mainly physical interactions. Increasing curcumin content lowered contact angle, tuned hydration, and modulated release: 1–2 wt % enabled slow, sustained delivery (∼20% at 9 h), whereas 3 wt % accelerated release (∼50% at 9 h) due to morphology-related effects. Water-vapor permeability was high initially but decreased markedly over 72 h as the fibrous network equilibrated. Orientation-dependent UV tests showed that the PEF layer effectively shielded the curcumin-loaded PLA from UV-A, while direct exposure of the PLA side caused measurable degradation. This structurally engineered, biobased bilayer couples mechanical reinforcement, barrier and UV-shielding with controllable payload release, supporting sustainable films for biomedical, food packaging, and other protective applications.

## Graphical abstract

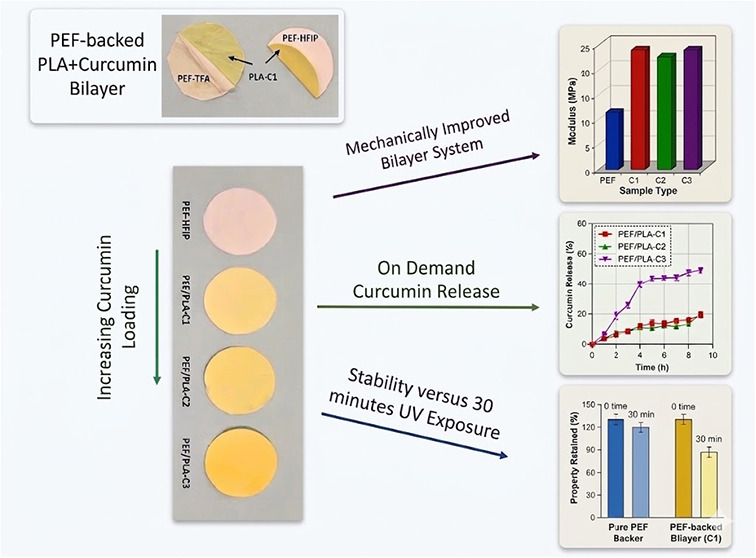

## 1 Introduction

Poly­(lactic acid) (PLA) is one of the most extensively explored biodegradable polymers for biomedical and packaging applications due to its renewable origin, biocompatibility, and regulatory acceptance.^1^ In biomedical contexts such as wound dressings, local drug-delivery systems, and protective interfaces, PLA electrospun films offer tunable porosity, high specific surface area, and processability into nanofibrous structures capable of hosting a wide range of therapeutic agents.^2^ Electrospinning is particularly attractive in this context because it enables precise control over fiber diameter and layer architecture, allowing fabrication of highly porous films with tailored structural and functional properties. Effective wound-care materials, for instance, must maintain a moist environment, control exudate, provide mechanical stability, and deliver active agents in a sustained manner, while also protecting them from environmental degradation such as moisture and light exposure. Similar functional requirements arise in active packaging and protective coating applications, where mechanical integrity, barrier control, and stability of incorporated actives are equally important.^3^

Despite its broad utility, PLA suffers from several intrinsic limitations that restrict its performance in advanced biomedical and functional applications. PLA is inherently brittle, with low elongation at break and poor impact strength, which can result in premature tearing, reduced handling stability, and limited conformability in applications requiring flexibility.^4,5^ Its hydrophobic character also leads to poor wettability and limited hydration, which can hinder interactions with aqueous environments in biomedical systems and reduce the efficiency of processes that rely on fluid absorption or diffusion.^2^ In addition, PLA has poor UV-shielding ability that further reduces its effectiveness in protecting sensitive compounds.^6,7^ These limitations are compounded by PLA’s relatively low thermal stability and slow crystallization rate, which can negatively affect processing, dimensional stability, and end-use performance.

To overcome these challenges, a wide range of strategies has been explored to enhance the mechanical, barrier, and functional characteristics of PLA-based films. Approaches such as polymer blending, addition of plasticizers, and incorporation of nanofillers have shown improvements in flexibility, toughness, and thermal behavior.^4^ Nanoparticle reinforcement and high-barrier polymer blends have also been used to reduce oxygen and water-vapor permeability by increasing pathway tortuosity or modifying the crystalline morphology. Similarly, surface modification techniques, including plasma treatment, have been employed to improve hydrophilicity, interfacial adhesion, and selective permeability in PLA.^8^ Among these approaches, bilayer and multilayer structures have emerged as particularly effective, as they allow different layers to contribute distinct functionalities. Bilayer systems such as PLA/chitosan films have demonstrated improved tensile strength, ductility, and barrier performance, highlighting the potential of multilayer structures to address PLA’s inherent shortcomings.^5^

Poly­(ethylene furanoate) (PEF) is a fully biobased polyester derived from 2,5-furandicarboxylic acid, attracting increasing interest as a sustainable alternative to PET. Although PEF exhibits superior barrier performance compared with PLA, it also suffers from intrinsic mechanical limitations, including brittleness and limited ductility, which can restrict its standalone application in flexible films. PEF exhibits a higher glass-transition temperature, high stiffness, and markedly reduced permeability to oxygen, carbon dioxide, and water vapor, owing to its rigid furan-ring backbone.^9^ To address these mechanical shortcomings, blending and hybridization strategies have been widely explored, with PEF-based blends such as PEF/PE^10^ and PEF/PLA^11^ demonstrating significantly improved mechanical durability and toughness. Similarly, electrospinning PEF in combination with other polymers, including PLA and PET, has been shown to enhance fiber integrity, flexibility, and overall performance. These findings highlight the importance of polymer–polymer interactions in mitigating the inherent brittleness of PEF while retaining its excellent barrier characteristics. In this study, we introduce a novel electrospun PLA–PEF bilayer system, where curcumin-loaded PLA forms the functional top layer and PEF serves as a mechanically supportive and barrier-enhancing backing layer. Curcumin was selected as the model bioactive compound due to its well-established antioxidant, anti-inflammatory, and antimicrobial properties, which make it a widely used functional additive in PLA-based electrospun films.^1,12,13^ We hypothesized that identifying a suitable solvent system for PEF would be essential for producing a stable backing layer with strong interfacial adhesion to PLA. Furthermore, integrating this optimized PEF layer beneath the PLA fibers was expected to enhance the overall functionality of the bilayer films, including improved mechanical performance, regulated hydration behavior, and superior photoprotection of the active layer. By establishing this bilayer configuration, the present work aims to overcome several intrinsic limitations of PLA and PEF by leveraging synergistic interactions between the two polymers, while supporting the use of sustainable, biobased materials in advanced film designs.

## 2 Materials and Methods

### 2.1 Materials

Poly­(lactic acid) (PLA, commercial grade 4032D) was purchased from NatureWorks LLC (Hangzhou, China). Poly­(ethylene furanoate) (PEF) was obtained from Avantium, Netherlands, with a molecular weight >30 000 g/mol. Curcumin (≥95% purity) was sourced from Belle Chemical (USA). Solvents including trifluoroacetic acid (TFA, 99%, Pcode: 102285924) and 1,1,1,3,3,3-hexafluoro-2-propanol (HFIP, ≥99%, Pcode: 102125868) were supplied by Sigma-Aldrich. Dichloromethane (DCM, analytical grade) and dimethylformamide (DMF, analytical grade) were obtained from Merck. Tween 80 was purchased from Sigma-Aldrich and used in release experiments. Phosphate-buffered saline (PBS, pH 7.4) was prepared using analytical-grade reagents and used for swelling, degradation, and drug-release studies. All materials and chemicals were used as received without further purification.

### 2.2 Fabrication of Electrospun Films

The electrospinning solutions were prepared according to the formulations listed in Table 1. For monolayer PEF films, PEF (8% w/v) was dissolved in 20 mL of either TFA or HFIP and stirred magnetically overnight at room temperature to ensure complete dissolution, in accordance with previously reported procedures with minor modifications.^11^ For monolayer PLA films, PLA (8% w/v) was dissolved in 18 mL of DCM and stirred overnight; immediately before electrospinning, 2 mL of DMF containing the required amount of curcumin (1–3% w/w relative to PLA, depending on the formulation) was added to reduce the risk of solvent-induced or light-induced degradation of curcumin. Electrospinning was carried out using an E-Fiber EF300 electrospinning unit (SKE Research Equipment, Italy) equipped with a 50 mL syringe and a 20G metallic spinneret, operating at an applied voltage of 22 kV, a solution flow rate of 5 mL h^–1^, and a rotating drum collector speed of 500 rpm.

**Table 1:** Composition and Solvent Systems Used for Preparing Electrospun Monolayer and Bilayer PEF–PLA Films^a^

Code	Sample description	PEF solution (8% w/v)–solvent, volume	PLA solution (8% w/v)–solvent, volume	Curcumin loading (% w/w relative to PLA)
PEF-TFA	PEF monolayer (TFA-based)	TFA, 40 mL	–	0
PEF-HFIP	PEF monolayer (HFIP-based)	HFIP, 40 mL	–	0
PLA-C1	PLA monolayer with 1% curcumin	–	DCM:DMF (9:1), 40 mL	1
PEF/PLA-C1	Bilayer: PEF backing + PLA with 1% curcumin	HFIP, 20 mL	DCM:DMF (9:1), 20 mL	1
PEF/PLA-C2	Bilayer: PEF backing + PLA with 2% curcumin	HFIP, 20 mL	DCM:DMF (9:1), 20 mL	2
PEF/PLA-C3	Bilayer: PEF backing + PLA with 3% curcumin	HFIP, 20 mL	DCM:DMF (9:1), 20 mL	3

a Polymer concentrations are expressed as % w/v in the corresponding solvent system, whereas curcumin loading is expressed as % w/w relative to PLA.

Bilayer films were produced by first electrospinning the PEF layer by using the same process conditions mentioned above. While the PEF mat remained on the rotating drum collector, the curcumin-loaded PLA solution was electrospun directly onto its surface to obtain a cohesive bilayer structure (see schematic in Figure 1). The same mixing procedure was followed for all curcumin loadings. The PLA monolayer (PLA-C1) incorporating 1% curcumin was fabricated and used as a control to compare with bilayer films.

**Figure 1:**
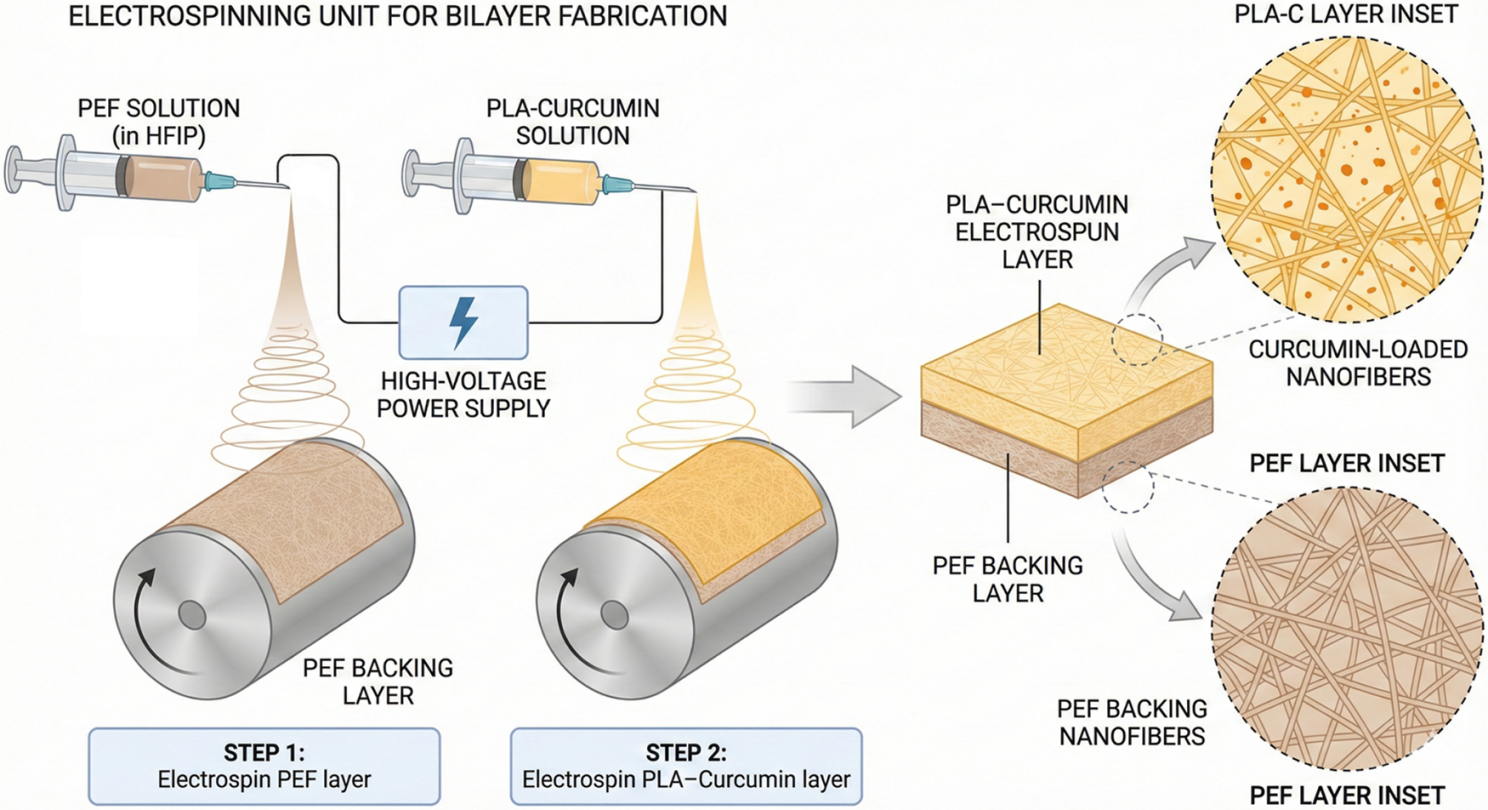
Schematic representation of the sequential electrospinning process used to fabricate the PEF/PLA-C bilayer, showing deposition of the PEF backing layer followed by the PLA-curcumin top layer and the resulting bilayer structure.

### 2.3 Characterization of Electrospun Films

#### 2.3.1 Morphology

The surface morphology and fiber size distribution of the electrospun films were investigated using a scanning electron microscope (SEM, JEOL JSM-5600) operated at an accelerating voltage of 10 kV. Prior to imaging, samples were coated with a thin layer (∼3–5 nm) of gold using a sputter coater (JEOL JFC-1200) for 30 s to enhance conductivity. SEM micrographs were analyzed with ImageJ software to determine the average fiber diameter.^14^ Measurements were carried out on 100 randomly selected fibers from each sample to ensure representative statistics.

#### 2.3.2 Mechanical Properties

The mechanical properties of the electrospun films were evaluated using a universal testing machine (UTM, Shimadzu Autograph AG-X, 10 kN load cell). Rectangular specimens were carefully cut from the membranes and mounted between the machine grips, ensuring proper alignment to avoid slippage or uneven loading. The crosshead speed was maintained at 1 mm/min throughout the test.^11^ Force displacement data were recorded and subsequently converted to stress–strain curves, from which tensile strength, elongation at break, and the Young’s modulus were determined. All measurements were performed at room temperature, and at least three samples were tested for each film to ensure reproducibility.

#### 2.3.3 FTIR Analysis

The bonding and molecular interactions within the electrospun films were investigated using an attenuated total reflectance Fourier transform infrared (ATR-FTIR) spectrometer (PerkinElmer, UATR Two). Spectra were recorded over 4000–400 cm^–1^ with automatic baseline correction, using 32 scans per sample. To assess the influence of curcumin incorporation and bilayer formation, spectra of pure curcumin and the PLA-C1 monolayer were first collected. These were then compared with spectra of the curcumin-loaded bilayer films (PEF/PLA-C1), enabling identification of characteristic functional groups and potential intermolecular interactions between components.

#### 2.3.4 Wettability

The wettability of the electrospun films was assessed using a customized contact angle setup, where the tangent of the droplet profile was measured with an integrated angle tool. A USB-based portable digital microscope equipped with LED illumination and operated through MicroCapture Plus software (version 1.01) was employed to capture images of water droplets placed on the film surface. Droplets were dispensed from a fixed height of 1 cm using a 22G needle (inner diameter: 0.406 mm) connected via Tygon tubing (1/16″ inner diameter) to a syringe pump (Fusion 200, Chemyx Inc., Stafford, US). The dispensing rate was maintained at 0.06 mL/min to ensure controlled droplet deposition.

#### 2.3.5 Swelling Ratio

The swelling behavior of electrospun films was evaluated in phosphate-buffered saline (PBS, pH 7.4) at 37 °C to simulate a physiological aqueous environment. Square samples (2 cm × 2 cm) of each film were immersed in 2.5 mL PBS for 24 h. The swelling ratio (SR, %) was determined gravimetrically by weighing the wet samples (*W_s_*) after gently removing excess surface moisture with Whatman filter paper and comparing them with their corresponding dry weights (*W_d_*) using the following equation^15^

1 $$\mathrm{SR}\;\left(\%\right)=\frac{W_{s}-W_{d}}{W_{d}}\times 100$$

#### 2.3.6 Water Vapor Permeability

The film’s water vapor barrier properties were evaluated using a modified gravimetric method. Each membrane (5 cm × 5 cm) was used to seal a test tube containing distilled water, which was incubated at 37 °C under a controlled ambient relative humidity of 50% RH inside the incubator. This configuration maintained an approximate internal water vapor pressure of 6.3 kPa (100% RH at 37 °C) and an external vapor pressure of 3.15 kPa (50% RH at 37 °C), resulting in a driving force (Δ*P*) of 3.15 kPa. This method was adopted for the intended application of the films on human skin, which naturally maintains a surface temperature of approximately 37 °C and near-saturation moisture conditions due to trans-epidermal water loss (TEWL), where 50% RH is more relevant than 0% RH.^16^ The mass loss of each tube was measured at set time intervals. The water vapor transmission rate (WVTR, gm^–2^ day^–1^) was calculated as follows

2 $$\mathrm{WVTR}=\frac{\Delta W}{A\cdot t}$$

where, Δ*W* is the weight loss of the sealed tube (g), *A* is the exposed film area (m^2^), and *t* is the time (days). Then, WVP (gm^–1^ s^–1^ Pa^–1^) was calculated by normalizing film thickness and the water vapor pressure difference across the film as follows

3 $$\mathrm{WVP}=\frac{\mathrm{WVTR}\cdot x}{\Delta P}$$

where, *x* is the film thickness (m), Δ*P* is the water vapor pressure difference between the inside test tube and outside the test tube, giving the value of ≈3.15 kPa.

#### 2.3.7 Curcumin Release Studies

The *in vitro* release of curcumin from the electrospun films (PLA-C1 and bilayer samples PEF/PLA-C1, PEF/PLA-C2, and PEF/PLA-C3) was evaluated in PBS buffer (pH 7.4) supplemented with 1% (v/v) Tween 80, a nonionic surfactant commonly used to solubilize hydrophobic compounds in release studies. Film specimens (4 cm × 4 cm) were placed in 50 mL test tubes containing 10 mL of the release medium. The tubes were kept in a thermostatic shaking incubator set to 37 °C and 50 rpm and were protected from light to minimize photodegradation of curcumin.

At predetermined time intervals (up to 10 h), a 360 μL aliquot of the release medium was withdrawn from each tube and immediately replaced with an equal volume of fresh prewarmed PBS/Tween 80 (37 °C) to maintain constant volume and approximate sink conditions. The withdrawn samples were analyzed using a UV–vis spectrophotometer (Epoch 2 Microplate Spectrophotometer, BioTek, USA) at 475 nm. Curcumin concentrations in the release samples were determined from a calibration curve constructed in PBS/Tween 80 over the range 0–60 μg/mL at 475 nm.

The total drug content in the films was determined separately using film specimens of the same dimensions (4 cm × 4 cm). Each specimen was dissolved in 2 mL of DMF, followed by centrifugation at 5000 rpm for 10 min and standing until the PLA phase was completely dissolved, releasing the curcumin, while the PEF fraction remained undissolved. The supernatant was analyzed at 475 nm, and the curcumin concentration was obtained from a calibration curve prepared in DMF over the range 0–50 μg/mL. From this, the total curcumin content per film specimen, *W_t_*, was calculated.

The cumulative percentage of curcumin released (CR, %) at each sampling time *n* was calculated using

4 $$\mathrm{CR}\;\left(\%\right)=\frac{V_{s}\sum_{i=1}^{n-1}C_{i}+C_{n}V_{t}}{W_{t}}\times 100$$

where *V_s_* is the volume of each withdrawn sample (mL), *C_i_* is the curcumin concentration (μg/mL) in the sample collected at time point *i*, *C_n_* is the concentration measured at the current time point *n*, *V_t_* is the total volume of the release medium in the tube (mL), and *W_t_* is the total curcumin content (μg) in the corresponding film specimen.

To elucidate the release mechanism of curcumin from the electrospun films, the cumulative release data expressed as CR(%) were fitted to commonly used kinetic models, including zero-order, first-order, Higuchi, and Korsmeyer-Peppas models. For kinetic analysis, CR(%) values were converted to fractional release (*M*_*t*_/*M*_∞_ = CR/100), where *M*_*t*_ is the cumulative amount of curcumin released at time *t* and *M*_∞_ corresponds to the total curcumin content in the film specimen (*W*_*t*_). The mathematical expressions of the models are given below.

$$\mathrm{Zero}\;\mathrm{order}:\frac{M_{t}}{M_{\infty }}=k_{0}t+C_{0}$$ 5

$$\mathrm{First}\;\mathrm{order}:\frac{M_{t}}{M_{\infty }}=1-\exp\left(-k_{1}t\right)$$ 6

7 $$\mathrm{Higuchi}:\frac{M_{t}}{M_{\infty }}=k_{H}t^{1/2}$$

8 $$\mathrm{Korsmeyer}\text{−}\mathrm{Peppas}:\frac{M_{t}}{M_{\infty }}=k_{\mathrm{KP}}t^{n}$$

where *k*_0_, *k*_1_, *k*_H_, and *k*_KP_ are the kinetic rate constants, *C*_0_ is the zero-order intercept, and *n* is the release exponent indicative of the dominant transport mechanism.

#### 2.3.8 Photoprotection by the Backing Layer

The photostability of curcumin incorporated into the electrospun bilayer films was evaluated under controlled UV-A irradiation at 365 nm to assess the protective role of the PEF backing layer against light-induced degradation of the active compound within the bilayer structure. Specifically, the study examined whether curcumin embedded within the PLA layer undergoes degradation upon UV-A exposure and whether the PEF layer can effectively shield the curcumin-containing PLA matrix from direct irradiation. These experiments were conducted using a UV-A LED chamber (DSXUV 150 mm × 150 mm, Shenzhen, China) with a rated power of 32 W. The film specimens (2 cm × 2 cm) of PEF/PLA-C1 were used for this assessment. During irradiation, the samples were placed at a fixed distance of 20 cm from the UV light source to ensure consistent exposure conditions. For each test, two identical film samples with comparable weights were prepared. In the first configuration, the films were positioned with the PLA-curcumin side facing the UV source to allow direct exposure. In the second configuration, the films were inverted such that the PEF backing layer faced the UV lamp, thereby shielding the PLA-curcumin layer from direct light exposure. All samples were irradiated for 30 min at room temperature.

Following UV exposure, both control (unexposed) and irradiated films were dissolved in 2 mL of DMF, and the remaining curcumin content was quantified spectrophotometrically at 475 nm as described in Section 2.3.7. The extent of photodegradation was determined by comparing the curcumin content before and after UV exposure.

#### 2.3.9 Statistical and Kinetic Analysis

All experiments were performed in triplicate unless otherwise stated, and results are reported as mean ± standard deviation. Descriptive statistical analysis and graphical data representation were carried out using GraphPad Prism 11 (GraphPad Software, USA). Statistical comparisons between multiple groups were conducted using one-way analysis of variance (ANOVA), with differences considered statistically significant at *p* < 0.05.

Kinetic model fitting of curcumin release data was performed by regression analysis using Python 3.12 executed in the Spyder 5.5 integrated development environment. Commonly used release models, including zero-order, first-order, Higuchi, and Korsmeyer–Peppas models, were applied. The goodness of fit was evaluated using the coefficient of determination (*R* ^2^) and root mean squared error (RMSE).

## 3 Results and Discussion

### 3.1 Morphology of Electrospun Membranes

The morphology and interfacial integrity of the electrospun membranes were first examined to identify a suitable solvent system for fabricating a stable PEF backing layer in the bilayer configuration. PEF backing layers fabricated using TFA exhibited poor interfacial adhesion with the subsequently deposited PLA layer (Figure 2k) and were therefore not pursued further. This behavior can be attributed to the pronounced hydrophilicity imparted to PEF during electrospinning from TFA. Previous studies have shown that TFA induces partial solvation and scission of aliphatic polyester chains, generating surface −OH and −COOH groups that render PEF electrospun membranes superhydrophilic, with water contact angles approaching 0° within seconds.^11^ Such excessive surface hydration weakens interfacial interactions and limits effective adhesion with the comparatively hydrophobic PLA layer. Consequently, HFIP was selected as the solvent for PEF, as it yielded mechanically stable fibers with improved interlayer adhesion, and all PEF backing layers used in subsequent experiments were fabricated using HFIP. In this case, the sequential electrospinning process resulted in a strongly integrated bilayer structure, where the PLA layer adhered firmly to the underlying PEF mat and could not be easily separated without damaging the fibrous network, indicating strong interfacial cohesion.

**Figure 2:**
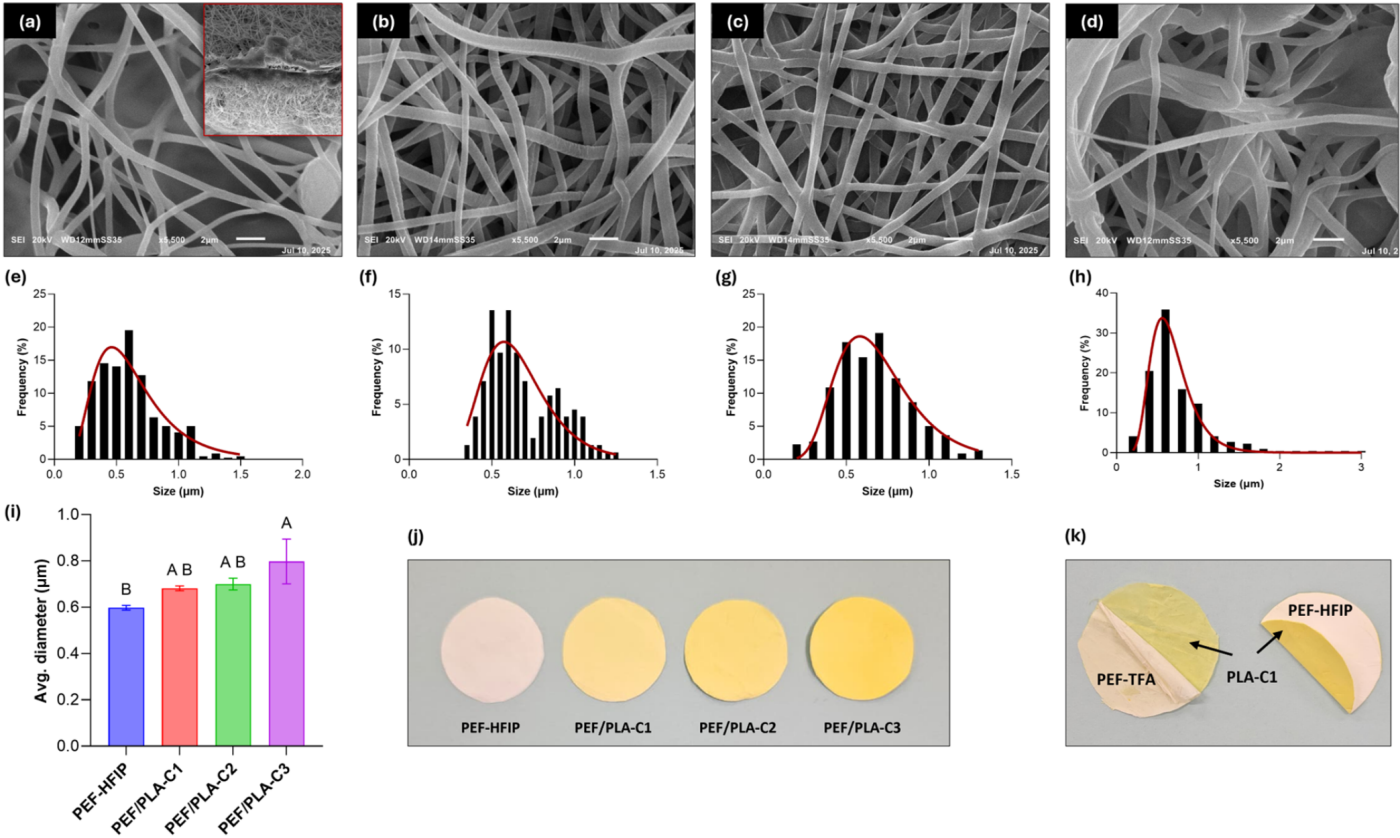
Morphological and physical characterization of electrospun membranes. (a) SEM micrograph of PEF-HFIP fibers; inset shows the cross-sectional view of the bilayer structure with PLA as the top layer and PEF as the backing layer. (b–d) SEM micrographs of PLA-based fibers and PEF–PLA bilayer membranes loaded with 1%, 2%, and 3% curcumin, respectively. (e–h) Corresponding fiber size distribution profiles. (i) Average fiber diameter of the different formulations (values with different letters indicate significant differences, *p* < 0.05). (j) Representative images of the fabricated films with PEF backing and increasing curcumin loadings. (k) Comparison of PEF backing layers prepared using HFIP and TFA solvents.

The morphology of PEF and curcumin-loaded PLA fibers and PEF–PLA bilayer membranes with different curcumin concentrations was examined by SEM (Figure 2a–d). The PEF-HFIP fibers (Figure 2a) exhibited relatively uniform and finer fiber diameters (∼0.6 μm), consistent with the higher chain rigidity associated with the furan-based polymer structure. PLA-C1 fibers (Figure 2b) exhibited relatively smooth, bead-free morphologies with slightly larger average diameters (∼0.68 μm). The bilayer PEF/PLA-C1 and PEF/PLA-C2 membranes (Figure 2c–d) showed comparable fibrous structures with average fiber diameters in the range of ∼0.68–0.70 μm, indicating no significant change at lower curcumin loadings. In contrast, the PEF/PLA-C3 sample exhibited a noticeable increase in average fiber diameter (∼0.80 μm) along with a broader size distribution, suggesting increased structural heterogeneity. At the highest curcumin loading (3%), regions of partial fiber stacking and fusion were observed, which can be attributed to curcumin-induced changes in solution properties. These changes can destabilize jet stretching and reduce solvent evaporation during electrospinning, promoting wet fiber deposition and interfiber adhesion. Similar morphological transitions have been reported for curcumin-loaded PCL systems, where higher drug content disrupts polymer chain packing and decreases crystallinity, resulting in fiber flattening and localized fusion upon collection.^17^ Additionally, increased curcumin loading can alter polymer-additive interactions and microstructural organization, which may promote localized phase heterogeneity and influence interfiber interactions during collection, as reported for curcumin-containing fibrous systems.^18^

Hence, the observed reduction in average fiber diameter in the bilayer membranes (Figure 2i) arises from a combined effect of (i) the presence of finer PEF fibers within the bilayer structure and (ii) the influence of curcumin on PLA fiber formation. Consistent with previous reports, PLA electrospun fibers typically exhibit diameters in the range of 0.5–2 μm, whereas PEF fibers commonly fall within 200–800 nm due to the higher chain rigidity imparted by the furan ring.^19,20^ Moreover, earlier studies have demonstrated that curcumin incorporation into PLA can further promote finer fiber formation through increased solution conductivity and possible plasticization effects, enhancing jet stretching during electrospinning.^21,22^ The macroscopic appearance of the electrospun films (Figure 2j) showed distinct color variations with increasing curcumin content, ranging from pale yellow at 1% loading to a deeper yellow at 3% loading, confirming incorporation of the bioactive compound. The thickness of the electrospun bilayer films was in the range of approximately 0.3 mm.

### 3.2 Mechanical Properties

The tensile strength, elongation at break, and Young’s modulus of the electrospun PLA-C1 monolayer and PEF/PLA-C (1–3) bilayer films are presented in Figure 3. The PLA-C1 film exhibited a tensile strength of 0.55 ± 0.03 MPa, an elongation at break of 43.1 ± 3.2%, and a Young’s modulus of 3.84 ± 0.90 MPa. Incorporation of a PEF backing layer resulted in a pronounced enhancement in mechanical performance for all curcumin-loaded bilayer films. Specifically, the bilayer films exhibited tensile strength in the range of 0.91–1.31 MPa, elongation at break of 100–123% (with higher variability at 3 wt % curcumin), and Young’s modulus in the range of 7.9–11.3 MPa. These enhancements are clearly reflected in the stress–strain curves, where the bilayer films exhibit substantially greater deformability than the PLA-C1 monolayer.

**Figure 3:**
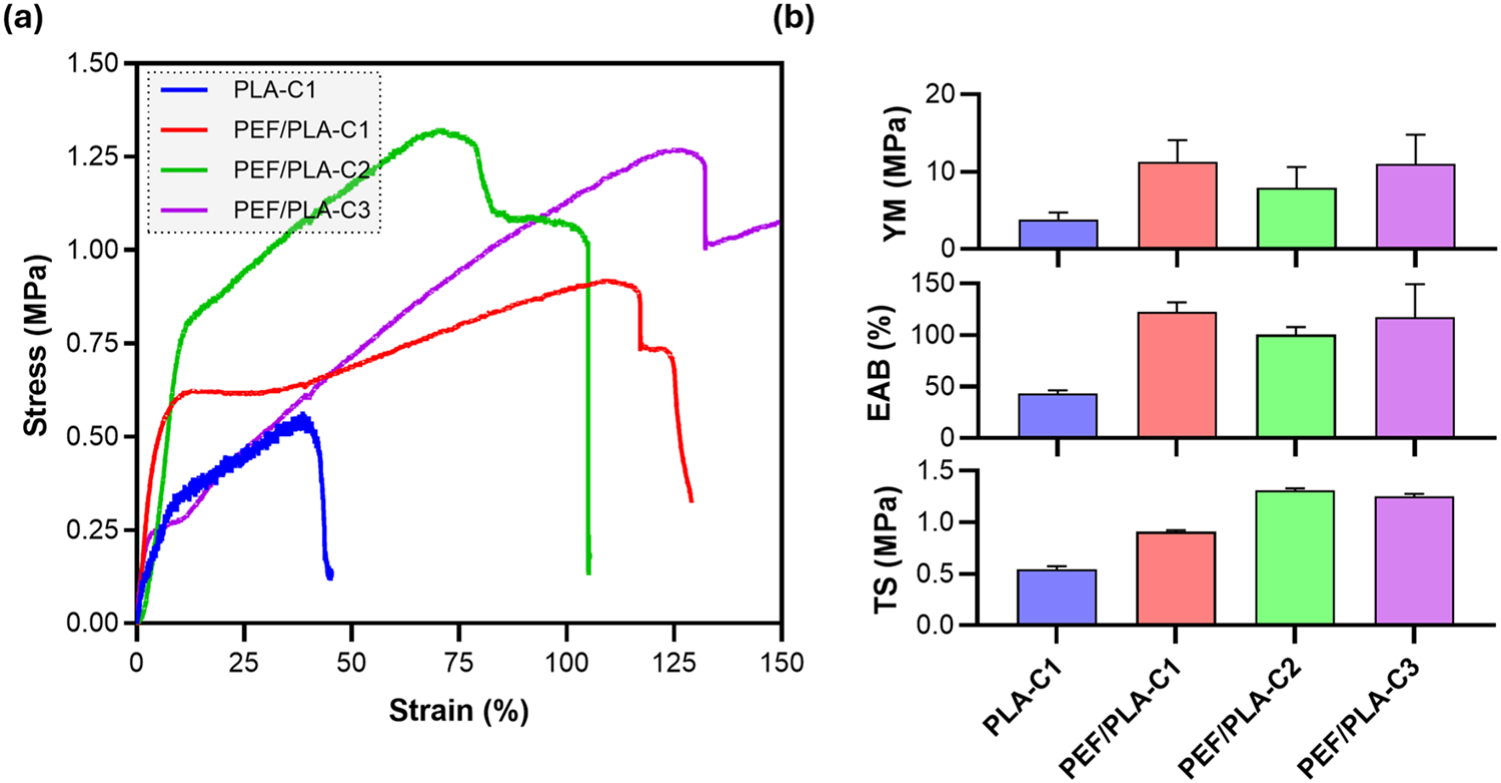
(a) Stress–strain curves of electrospun PLA-C1 monolayer and PEF/PLA-C bilayer films with different curcumin loadings, and (b) corresponding tensile strength (TS), elongation at break (EAB), Young’s modulus (YM) derived from the stress–strain data. Error bars represent standard deviation.

The incorporation of a PEF backing layer resulted in a clear improvement in the mechanical performance of the electrospun membranes, as reflected by higher tensile strength and elongation at break compared with the PLA-C1 monolayer. The relatively low strength and limited ductility of the PLA-C1 film are consistent with the brittle mechanical behavior typically observed in electrospun PLA mats, which is commonly attributed to their low intrinsic toughness and limited stress transfer between fibers.^23^ In the bilayer configuration, the presence of the PEF layer provides additional mechanical support, leading to improved stress distribution and delayed failure of the curcumin-loaded PLA layer. Similar enhancements in mechanical performance have been reported for PLA-based bilayer films, where the introduction of a mechanically supportive second layer improved stress transfer and delayed failure.^5^

In addition to the contribution of the PEF backing, curcumin loading in the PLA layer appears to influence the mechanical response in a concentration-dependent manner. Previous studies have shown that the effect of curcumin on the mechanical properties of electrospun fibers depends strongly on the polymer matrix and formulation. For example, in aliphatic polyesters such as PCL, moderate curcumin incorporation has been reported to improve tensile strength and elongation, whereas higher loadings result in reduced mechanical performance due to excessive plasticization and disruption of polymer chain packing.^17^ Similar nonmonotonic behavior has been observed in cellulose acetate nanofibers, where tensile strength increased up to a certain curcumin content and decreased at higher concentrations.^24^ By contrast, polyurethane-based electrospun mats have shown more sustained improvements in extensibility and strength upon curcumin addition, reflecting differences in polymer flexibility and intermolecular interactions.^25^

Within the present bilayer system, tensile strength increased with curcumin loading up to 2 wt % and remained comparable at 3 wt %, while elongation at break and Young’s modulus showed some variation within the range of experimental uncertainty. The observed mechanical response is therefore likely governed by the combined effects of the supporting PEF layer and modest modifications to the PLA fiber structure associated with curcumin incorporation. These results are consistent with prior reports indicating that moderate curcumin loadings can be accommodated in electrospun polymeric fibers without compromising mechanical performance, while higher concentrations may lead to deterioration depending on the polymer system.^17,24–26^

### 3.3 Chemical Structure and Intermolecular Interactions

The FTIR spectra of pure curcumin, neat PLA, and curcumin-loaded PLA (PLA-C1) are shown in Figure 4. Peaks at 1626 and 1505 cm^–1^ show carbonyl and ethylene groups in curcumin, respectively. The −C–O elongation of the phenolic group (−OH group) of curcumin was observed in the range of 1400–1500 cm^–1^.^27^ The peak at 1273 cm^–1^ indicated C–O stretch in ethers and peaks at 961, 807, 715 cm^–1^ were attributed to C–H bending in alkenes.^28^ Upon incorporation of curcumin, the PLA matrix in the PLA-C1 sample retained all the characteristic absorption bands observed in neat PLA.^29,30^ There was no significant change in frequency of the peaks in PLA due to addition of curcumin, indicating that there is no chemical interaction at structural level observed between PLA and curcumin.^13^ However, slight changes in relative contributions of amorphous and crystalline peaks,^29^ and a lower intensity C=O stretch in PLA-C1 compared to neat PLA indicate physical chain structure might have perturbed, which can be attributed to the hydrogen bonding between C=O in PLA with the −OH in curcumin (not shown here). Since no new bonds were observed, Rizehbandi et al.^31^ recently reported using Density Functional Theory combined with Machine Learning that there are electrostatic interactions and hydrogen bonding between the electron-rich and electron-poor regions of the chemical structures of curcumin and PLA. These physical interactions might be contributing toward the retarded release (less than 100%) of curcumin in the release study (explained later).

**Figure 4:**
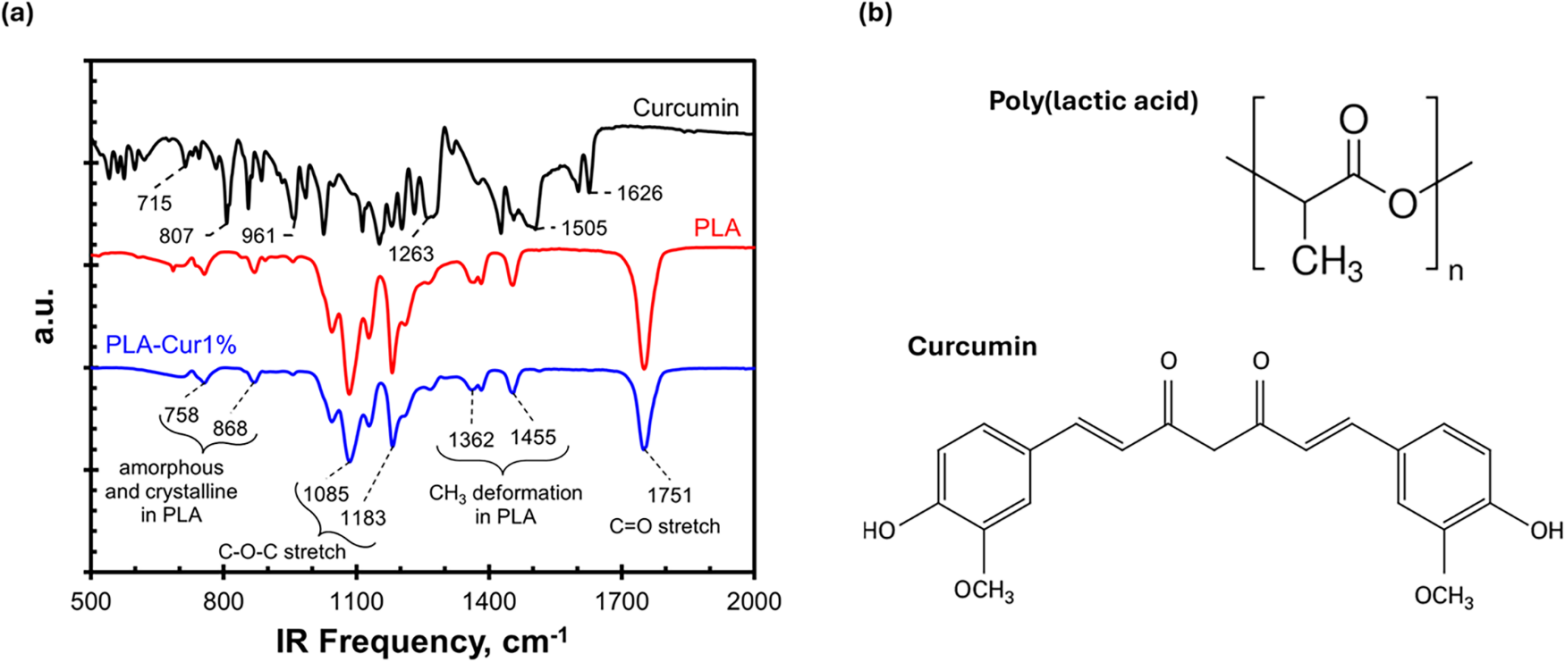
(a) FTIR spectra of pure curcumin, neat PLA, and curcumin-loaded PLA (PLA-C1), presented in arbitrary units (a.u.). (b) Chemical structures of the PLA repeating unit and curcumin.

### 3.4 Surface Wettability, Hydration, and Barrier Properties of Bilayers

The surface wettability of the electrospun bilayer membranes, evaluated by static water contact angle measurements (Figure 5a), reflects the combined influence of surface chemistry and fibrous architecture. Among the bilayer systems, a clear dependence of wettability on curcumin loading was observed. The PEF/PLA-C1 bilayer exhibited an average water contact angle of 103.1 ± 2.7°, indicating relatively hydrophobic surface behavior at low curcumin content. With increasing curcumin concentration, the bilayer membranes showed a progressive decrease in water contact angle. The contact angle decreased to 98.5 ± 3.6° for PEF/PLA-C2 and more markedly to 63.5 ± 10.4° for PEF/PLA-C3, indicating a substantial increase in surface wettability at higher curcumin loadings. Similar concentration-dependent reductions in contact angle have been widely reported for curcumin-loaded electrospun systems, particularly in PCL-based fibers, where curcumin incorporation progressively enhanced hydrophilicity.^32^ This behavior has been linked to the presence of oxygen-containing functional groups in curcumin (Figure 4b), which increase the polar component of surface energy and facilitate hydrogen bonding with water, despite curcumin’s limited aqueous solubility. Comparable effects have also been reported in CA/PCL nanofibers and related blended systems, where polymer–curcumin interactions promoted increased water affinity.^33^

**Figure 5:**
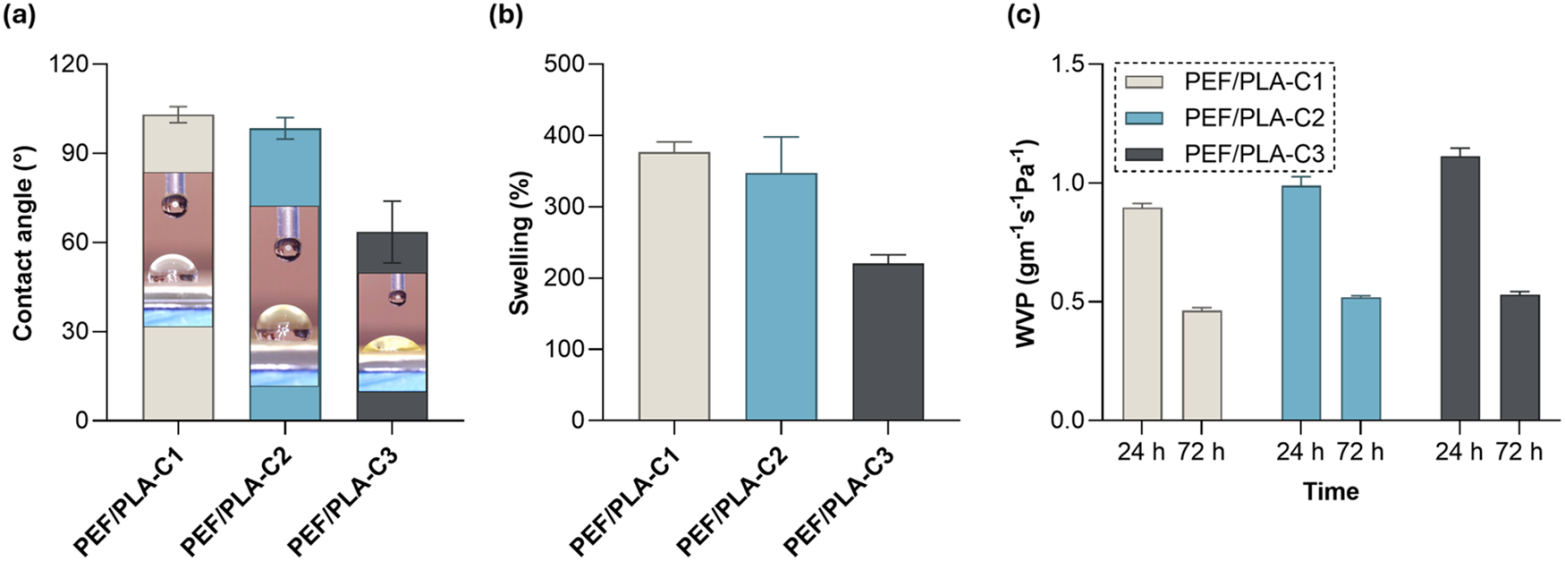
Physicochemical surface and barrier properties of electrospun films: (a) static water contact angle, (b) swelling behavior measured after 24 h, and (c) water vapor permeability (WVP) measured at 24 and 72 h. The error bars represent standard deviation from triplicate measurements.

In parallel with compositional effects, morphological changes associated with curcumin loading are expected to contribute to the observed wettability trends in the bilayer membranes. As discussed in Section 3.1, SEM micrographs (Figure 2b–d) show that pronounced variations in fiber diameter, packing density, and localized fiber fusion become evident at the highest curcumin loading (i.e., 3%), whereas such features are less prominent at lower concentrations. The influence of these microstructural parameters on the apparent contact angle of electrospun mats is well established. Highly porous and rough fibrous networks can exhibit elevated apparent contact angles due to air trapping at the solid–liquid interface, whereas partial fiber fusion or reduced surface roughness can facilitate liquid penetration into the fibrous network and lead to lower contact angles.^34–36^

The swelling behavior of the electrospun bilayer membranes was evaluated after 24 h of immersion to assess their equilibrium hydration response (Figure 5b). All bilayer samples exhibited high swelling ratios, indicating substantial bulk water uptake typical of electrospun fibrous systems. Among the bilayers, PEF/PLA-C1 showed a high swelling response, with values of approximately 367–387% after 24 h. The PEF/PLA-C2 bilayer exhibited comparable swelling, ranging from approximately 313–383%, indicating that intermediate curcumin loading maintains a high hydration capacity. In contrast, PEF/PLA-C3 displayed a markedly reduced swelling response, with values of approximately 212–229%, remaining significantly lower than those of PEF/PLA-C1 and PEF/PLA-C2. The reduced swelling observed for PEF/PLA-C3 correlates with the distinct morphology identified for this composition (Figure 2d), where localized fiber fusion and stacking lead to a denser fibrous structure. Similar morphology-related reductions in swelling have been reported for electrospun mats in which fiber fusion or clustering decreases accessible pore volume and limits bulk water uptake.^37^ These findings indicate that, within the bilayer system, curcumin loading influences swelling primarily through morphology-associated changes rather than curcumin content alone.

Water vapor permeability (WVP) of the electrospun bilayer membranes varied with curcumin loading and decreased with exposure time (Figure 5c). At 24 h, the bilayer films exhibited relatively high permeability, with WVP values of approximately 0.91, 1.02, and 1.09 g·m^–1^·s^–1^·Pa^–1^ for PEF/PLA-C1, PEF/PLA-C2, and PEF/PLA-C3, respectively. A statistically significant difference was observed among the films at this time point (*p* = 0.014), with the primary difference occurring between PEF/PLA-C1 and PEF/PLA-C3, while PEF/PLA-C2 showed intermediate behavior. This indicates that curcumin loading has a limited effect on WVP at lower concentrations, whereas at higher loading levels the associated morphological changes begin to influence vapor transport. As observed in the SEM images (Figure 2b–d), PEF/PLA-C1 and PEF/PLA-C2 exhibit relatively similar fibrous structures, whereas PEF/PLA-C3 shows a less uniform network with partial fiber fusion and larger accessible void regions, which may facilitate vapor diffusion. WVP appears to be influenced primarily by fibrous morphology, with polymer chemistry (i.e., curcumin content) playing an indirect role through its effect on fiber formation and microstructural features. Highly porous fibrous networks provide interconnected pathways that facilitate vapor diffusion, whereas localized densification or reduced pore accessibility can restrict transport.^38,39^ With increasing exposure time, WVP decreased for all bilayer samples, reaching approximately 0.47–0.54 g·m^–1^·s^–1^·Pa^–1^ after 72 h, indicating a clear time-dependent reduction in vapor transport. This reduction is consistent with the swelling behavior discussed above, where water uptake by the fibrous network leads to partial occupation of pore space and reduced effective diffusion pathways.^39,40^

The observed trends in wettability, swelling, and water vapor permeability reflect the combined influence of surface chemistry and fibrous structure in the electrospun membranes. Wettability is affected by both curcumin incorporation and morphological features, while changes in swelling and WVP are likely related to bulk structural characteristics associated with the membrane structure.

### 3.5 *In Vitro* Curcumin Release from Bilayers

Figure 6 presents the cumulative release profiles of curcumin from the PEF/PLA-C (1–3) bilayer films over a 9 h period. The bilayer films exhibited release behavior that was strongly dependent on curcumin loading, with pronounced differences observed at the highest curcumin content. The PEF/PLA-C1 and PEF/PLA-C2 bilayer films showed comparable release profiles, with cumulative release reaching approximately 20.5% and 21.3% at 9 h, corresponding to an estimated average release rate of ∼2.28%·h^–1^. In contrast, the PEF/PLA-C3 bilayer film exhibited a markedly higher release, reaching approximately 50.3% by 9 h, corresponding to an increased release rate of ∼5.59%·h^–1^.

**Figure 6:**
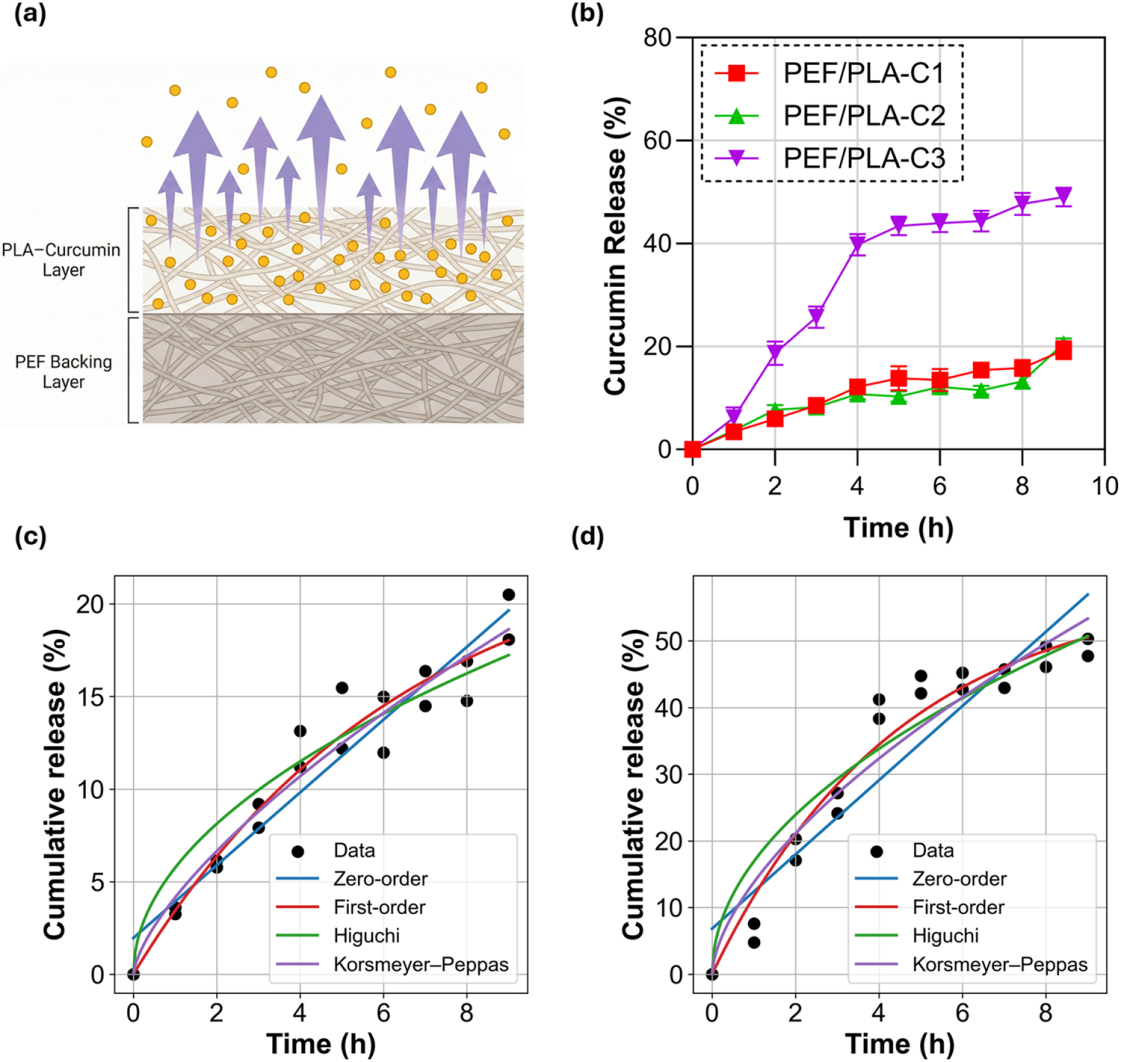
Curcumin release behavior and kinetic analysis of electrospun PEF/PLA bilayer films: (a) schematic illustration of directional curcumin release from the curcumin-loaded PLA layer in the bilayer structure [image generated using AI], (b) experimental cumulative release profiles of PEF/PLA-C bilayer films with different curcumin loadings, and kinetic model fitting of release data for (c) PEF/PLA-C1 (1% curcumin) and (d) PEF/PLA-C3 (3% curcumin) using zero-order, first-order, Higuchi, and Korsmeyer–Peppas models.

Within the bilayer system, the influence of curcumin concentration on release behavior became pronounced only at the highest loading level. While PEF/PLA-C1 and PEF/PLA-C2 displayed similar release profiles over the 9 h period, PEF/PLA-C3 exhibited a significantly accelerated release, approaching ∼50% cumulative release within the experimental time frame. This nonlinear increase in release at 3 wt % curcumin is consistent with reports indicating that higher curcumin loadings can disrupt polymer chain packing, generate microvoids, and increase surface roughness, thereby enhancing diffusive transport.^41^ This interpretation is further supported by the wettability results, where PEF/PLA-C3 exhibited a substantially lower contact angle, indicating enhanced surface hydrophilicity. Increased exposure of curcumin’s phenolic −OH groups at the fiber surface and partial phase separation at higher loading likely promote faster wetting and accelerated hydration relative to lower-curcumin bilayer formulations.

Kinetic modeling of curcumin release from the electrospun PEF/PLA bilayer films revealed a clear dependence of release behavior on curcumin loading (Figure 6c–d). For the low-loading bilayer (PEF/PLA-C1), the release data were well described by all tested models, with the first-order model providing the best fit (*R* ^2^ ≈ 0.95), indicating concentration-dependent release governed primarily by diffusion through the PLA fibrous matrix. For the high-loading bilayer (PEF/PLA-C3), the first-order model again yielded the highest correlation coefficient (*R* ^2^ ≈ 0.96), whereas the zero-order and diffusion-based models showed lower agreement. This behavior reflects a more strongly nonlinear release profile at elevated curcumin content, where an initial contribution from curcumin located near the fiber surface is followed by diffusion-limited release of partially entrapped curcumin within the PLA fiber interior; together with localized physical interactions between curcumin and the PLA matrix, this leads to time-dependent diffusivity rather than a constant release rate. Similar deviations from ideal diffusion-controlled behavior, attributed to combined diffusion and matrix-controlled transport, have been reported for curcumin-loaded electrospun poly­(DL-lactic-*co*-glycolic) acid (PLGA) systems.^42^

### 3.6 Photoprotection by the PEF Backing Layer

Figure 7 shows the amount of curcumin remaining in the bilayer films following UV exposure applied to either the PEF-facing or PLA-facing surface of the PEF/PLA-C1 bilayer. When irradiated from the PEF side, the curcumin content remained essentially unchanged, with mean values of 26.75 μg and 26.72 μg measured at 0 and 30 min, respectively. In contrast, when the PLA-curcumin side was directly exposed to UV light, the curcumin content decreased from 38.64 μg at 0 min to 35.55 μg after 30 min of irradiation. Comparison of percent degradation between the two film orientations revealed a significant difference (∼8% compared to initial value), demonstrating the superior photoprotective effect of the PEF backing layer.

**Figure 7:**
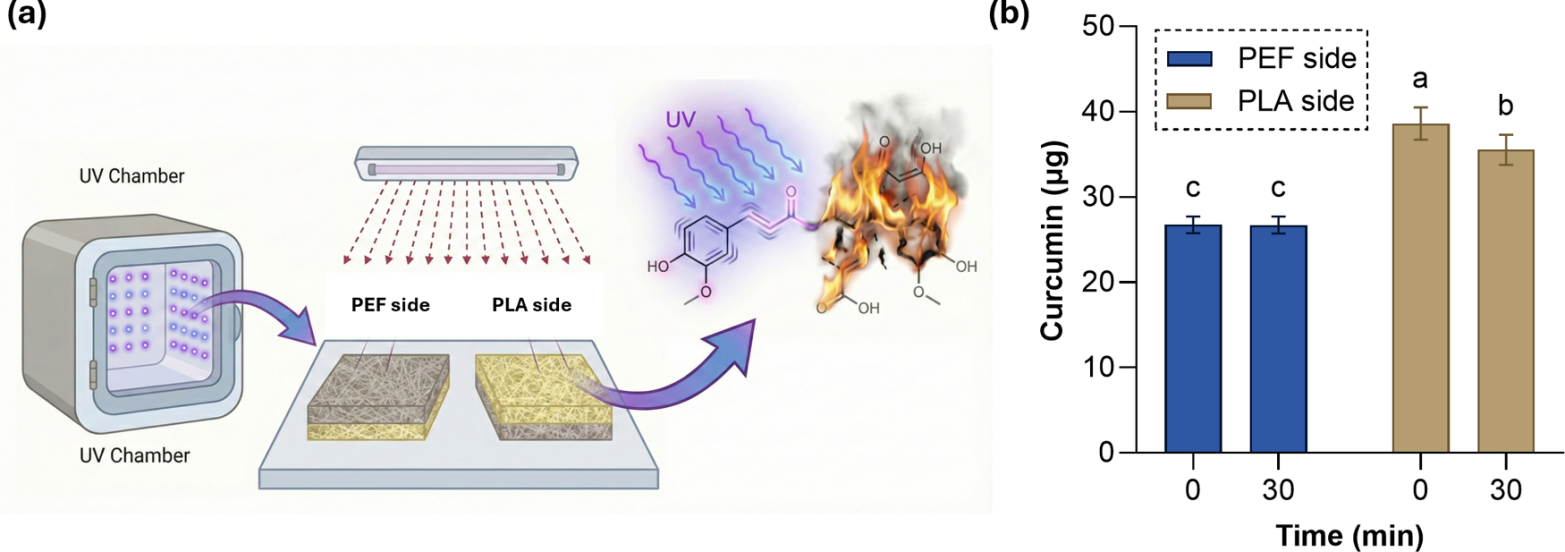
Evaluation of photoprotection in PEF/PLA bilayer films: (a) schematic illustration of the UV-A exposure setup showing film orientation with either the PEF-facing or PLA-facing side exposed to the UV source (image generated using AI), and (b) remaining curcumin content in the bilayer films before and after 30 min of UV exposure for the two exposure orientations. The error bars represent standard deviation, and different letters indicate statistically significant differences (*p* < 0.05).

Curcumin is known to undergo rapid photolysis under UV radiation due to cleavage of its conjugated structure and therefore requires effective shielding to maintain stability.^43^ Although curcumin is embedded within the PLA fibers, the present results indicate that this encapsulation alone is insufficient to prevent photodegradation. While electrospun fibrous networks can scatter incident light and reduce transmission to some extent,^44^ PLA-C1 films provide limited intrinsic UV protection. The pure PLA films are reported to exhibit high UV transmittance in both the UVB (280–320 nm) and UVA (320–400 nm) ranges, with transmittance values of approximately 60.8% and 35.2%, respectively, and a low ultraviolet protection factor (UPF ≈ 3.1), rendering them ineffective as UV barriers without modification.^45^ Prolonged UV exposure can further induce photoaging, molecular weight reduction, and embrittlement of PLA, limiting its performance under light exposure.^43^ Various strategies have been explored to improve the UV-shielding capability of PLA-based films, including the incorporation of inorganic fillers and functional additives. For example, ZnO nanoparticles have been shown to markedly enhance UV-blocking efficiency in electrospun PLA fibers, even at low loadings,^46^ while chitosan-derived additives have been reported to increase the UPF of PLA films from 0.45 to 34.45.^47^ In contrast to such compositional modifications, the present bilayer approach relies on structural design, where the curcumin-loaded PLA layer functions as the active reservoir and the PEF layer serves as an external physical barrier.

In the PEF/PLA-C1 bilayer configuration, orientation-dependent UV exposure clearly demonstrates the protective role of the PEF backing. When the PEF layer faces the light source, it acts as a dense shielding layer that limits light penetration and effectively suppresses curcumin photodegradation. Conversely, direct exposure of the PLA-curcumin surface leads to increased light transmission and accelerated degradation. Thus, positioning the PEF layer on the external side enables preferential release from the PLA layer while simultaneously protecting the active compound from environmental UV stress. This directional protection highlights the dual functionality of the PEF backing layer, combining mechanical support with effective photoprotection, and highlights the advantage of bilayer electrospun architectures for applications requiring controlled release and enhanced environmental stability.

## 4 Conclusion

This work shows that designing PEF/PLA electrospun films in a bilayer configuration is an effective way to combine the strengths of both polymers while minimizing their individual limitations. Using PEF as a backing layer provided clear improvements in mechanical stability and barrier performance, while the PLA layer served as an efficient carrier for curcumin without the need for chemical modification. The bilayer structure allowed surface wettability, hydration, and release behavior to be tuned through curcumin loading, with higher concentrations influencing fiber morphology and accelerating release. Importantly, the PEF layer also acted as a protective shield against UV exposure, significantly reducing photodegradation of the active compound. These findings show that a structurally engineered bilayer approach can deliver multifunctional performance and offer a practical route for developing sustainable electrospun films for biomedical, food packaging, and related protective applications. However, the present study is limited to laboratory-scale fabrication, and further work is required to address scalability of sequential electrospinning, including adaptation to high-throughput or continuous manufacturing processes.
